# Mechano-Chemical Synthesis, Structural Features and Optical Gap of Hybrid CH_3_NH_3_CdBr_3_ Perovskite

**DOI:** 10.3390/ma14206039

**Published:** 2021-10-13

**Authors:** José Antonio Alonso, Paula Kayser, Bo-Kyung Hong, María Consuelo Álvarez-Galván, Francois Fauth, Carlos Alberto López

**Affiliations:** 1 Instituto de Ciencia de Materiales de Madrid, Consejo Superior de Investigaciones Científicas, Cantoblanco, 28049 Madrid, Spain; Pakayser@ucm.es (P.K.); sally.bkhong@ies.upm.es (B.-K.H.); 2 Departamenton de Química Inorgánica, Facultad de Ciencias Químicas, Universidad Compluense de Madrid, 28040 Madrid, Spain; 3 Instituto de Catálisis y Petroleoquímica, Consejo Superior de Investigaciones Científicas, Cantoblanco, 28049 Madrid, Spain; c.alvarez@icp.csic.es; 4 CELLS—ALBA Synchrotron Light Facility, Cerdanyola del Valles, E-08290 Barcelona, Spain; ffauth@cells.es; 5 Instituto de Investigación en Tecnología Química (UNSL-CONICET), Facultad de Química, Bioquímica y Farmacia, Universidad Nacional de San Luis, Almirante Brown 1455, San Luis 5700, Argentina

**Keywords:** methylammonium cadmium tribromide, acentric crystal structure, polar CH_3_NH_3_^+^ orientation, ultraviolet pigment and solar cells

## Abstract

Hybrid methyl-ammonium (MA:CH_3_NH_3_^+^) lead halide MAPbX_3_ (X = halogen) perovskites exhibit an attractive optoelectronic performance that can be applied to the next generation of solar cells. To extend the field of interest of these hybrid materials, we describe the synthesis by a solvent-free ball-milling procedure, yielding a well crystallized, pure and moisture stable specimen of the Cd tribromide counterpart, MACdBr_3_, which contains chains of face-sharing CdBr_6_ octahedra in a framework defined in the *Cmc*2_1_ (No 36) space group. The details of the structural arrangement at 295 K have been investigated by high angular resolution synchrotron x-ray diffraction (SXRD), including the orientation of the organic MA units, which are roughly aligned along the c direction, given the acentric nature of the space group. UV-vis spectra unveil a gap of 4.6 eV, which could be useful for ultraviolet detectors.

## 1. Introduction

Hybrid organic-inorganic halide perovskites are suitable as light absorbers in solar cells [1,2,3,4], with an efficiency of power conversion (PCE) of about 23%, similar to silicon-based devices. In particular, methyl-ammonium lead iodide CH_3_NH_3_PbI_3_ (CH_3_NH_3_^+^: MA) is the prototype of light harvester in hetero-junction solar cells [5,6,7]. Hybrid perovskites MAPbX_3_ (X = I, Br and Cl) also exhibit properties such as ambipolar charge mobility, low exciton binding energy and tolerance to structural defects [8,9,10,11,12,13,14]. Other divalent elements instead of Pb^2+^ have been evaluated; experimental [15] and theoretical [16,17] studies have been carried out for other divalent cations, such as those of Group 12 (Zn, Cd and Hg) that also are closed-shell divalent cations. However, structural and electronic studies on these materials are scarce. In particular, the Cd-containing phase MACdBr_3_ is little studied with only structural and theoretical analysis [17,18,19]. The addition of Cd^2+^ is paradigmatic since it is responsible for interesting phenomena; the electroluminescence is not inhibited, while a composite with polystyrene is effective in preventing the degradation of cadmium halide due to humidity [20].

The crystal structure of MACdBr_3_ differs from those of MAPbX_3_ (X = I, Br and I), most of which can be described in the cubic arystotype perovskite structure, defined in the space group *Pm*$\bar{3}$*m*. Given the smaller size of Cd^2+^ (0.95 Å) vs. Pb^2+^ (1.19 Å), the tolerance factor of the hypothetical perovskite would be higher than unity, and therefore, instead of the classical framework of corner-sharing octahedra, this Cd compound presents chains of face-sharing CdBr_6_ octahedra in a quasi 1D arrangement. It was described by Hassen et al. [19] from a single crystal prepared by solution chemistry. Additionally, Kallel et al. [18] described a phase transition at 170 K from the above described structure to a complex orthorhombic unit cell. Regarding the absorption properties, theoretical calculations in an unrealistic cubic structure yields a gap of 1.3 eV [17]. The experimental optical gap has not been reported in the literature.

In this paper, we describe the preparation of MACdBr_3_ by an alternative mechano-synthesis procedure, with green credentials since the utilization of organic solvents is not required. A sample with an excellent crystallinity was obtained by ball-milling under N_2_ atmosphere, characterized by laboratory x-ray diffraction (XRD), and the crystal structure was refined from synchrotron x-ray diffraction (SXRD) data. We propose the H positions, unveiling the H-bond interactions between the CH_3_NH_3_^+^ units and CdBr_6_ octahedral chains, where the particular distribution of ADP parameters is a result of N–H···Br interactions. The diffuse reflectance spectra of the sample were measured for the first time, observing a band gap of ~4.6 eV, shifted to the UV region with respect to the lead-containing MAPbBr_3_ counterpart.

## 2. Materials and Methods

MACdBr_3_ was prepared in polycrystalline form by ball milling (mechano-chemical synthesis) starting from stoichiometric proportions of CdBr_2_ and MABr. The mixture of bromides (totalling 1.5 g), was set into a zirconia-lined jar together with 30 zirconia balls (5 mm diameter) and sealed in a N_2_-filled glove box. The mechanically activated reaction was carried out in a Retsch (Haan, Germany) PM100 mill for 4 h at 400 rpm. Laboratory XRD was used for assessing phase purity; the XRD patterns were recorded in a Bruker (Germany) D5 diffractometer with K_α_Cu (λ = 1.5418 Å) radiation; the 2θ range was 4° up to 90° with increments of 0.05°.

The crystal structure of MACdBr_3_ was investigated at RT (295 K) by synchrotron X-ray powder diffraction (SXRD) using the MSPD station at the ALBA facility, Barcelona (Spain). Radiation with 38 keV energy, λ = 0.32511 Å, was selected in the high angular resolution mode (MAD set-up) [21]. The sample was measured in a glass capillary of 0.7 mm diameter, which rotated during data acquisition. The structural refinement by the Rietveld method [22] was carried out with the *Fullprof* software [23]. The full refinement of the profiles included the zero-point error; scale factor; background coefficients; unit-cell parameters; pseudo-Voigt shape parameters; atomic coordinates; anisotropic displacements for the metal and halogen atoms and isotropic for C and N from the methyl-ammonium groups.

Field-effect scanning electron microscopy (FE-SEM) pictures were collected on an FEI-Nova microscope, with an acceleration potential of 5 kV. The optical diffuse-reflectance spectrum was recorded at room temperature in a UV-vis spectrophotometer Varian Cary 5000. A photodetector device was fabricated by drop-casting the phase solution in dimetilformamide onto Au/Cr pre-patterned electrodes with a gap of 10 µm, and drying in a hot plate at 100 °C. The illumination power was 20 μW.

## 3. Results and Discussions

### 3.1. Initial Characterization: FE-SEM

**Figure 1:**
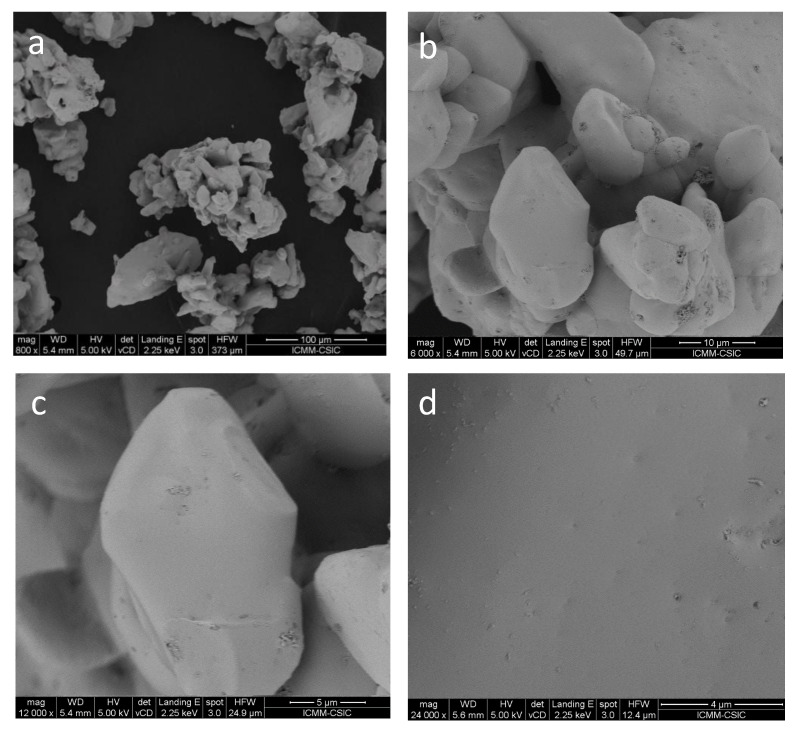
FE-SEM images of the MACdBr_3_ collected with magnifications of (**a**) 800×, (**b**) 6000×, (**c**) 12,000× and (**d**) 24,000×.

The hybrid CH_3_NH_3_CdBr_3_ compound was obtained as a white polycrystalline material. High-resolution FE-SEM images were obtained to get an insight into the microstructure of this product obtained by ball milling (Figure 1). Figure 1a illustrates an overall view with low magnification (800×), showing irregular-shaped clusters of particles of different sizes. Figure 1b,c unveil the presence of microcrystals of uneven form, with flat facets in the micrometer-size range (e.g., the crystal in Figure 1c has a width of 13 µm), which are grown during the ball milling process. Figure 1d illustrates the homogeneity of the crystals, with no particular microstructural features, in a large magnification view (24,000×). EDX analysis coupled to the FE-SEM images yields an atomic composition close to 1:3 for the Cd/Br ratio. A typical EDX spectrum is included in Figure S1 in the Supplementary Information; other SEM images are included in Figure S2. 

### 3.2. Structural Characterization

High angular resolution SXRD data allowed us a precise structural characterization. This is essential to accurately define the symmetry and crystallographic features. The pattern can be indexed in an orthorhombic unit cell with *a* = 7.91722(5) Å, *b* = 13.7108(1) Å, *c* = 6.89374(2) Å, and the crystal structure can be defined in the acentric *Cmc*2_1_ (No 36) space group, confirming the work by Ben Hassen et al. from single-crystal x-ray diffraction [19].

**Figure 2:**
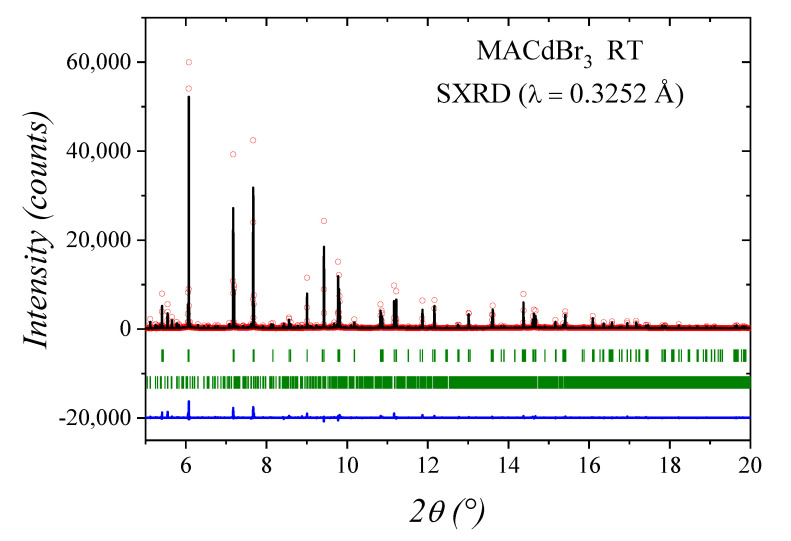
SXRD profiles for CH_3_NH_3_CdBr_3_ at RT. The experimental points are represented by red circles, while calculated profile is a black full line and the blue lower line is the difference. The first series of green markers correspond to the allowed Bragg positions for the main phase (*Cmc*2_1_ space group). The second series of markers corresponds to a minor impurity MA_2_CdBr_4_, (*P*2_1_/*c* space group).

**Figure 3:**
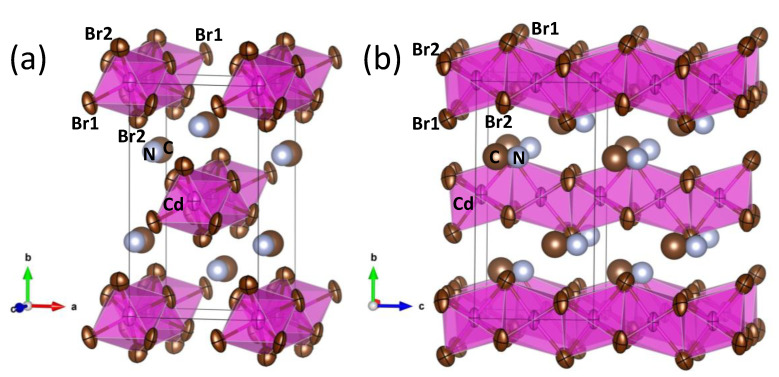
Two views of the crystal structure, showing the anisotropic atomic displacement parameters (ADP) of Br site and the face-sharing octahedral chains along c axis. (**a**) along c-axis direction, (**b**) along a-axis direction.

Cd^2+^ cations are allocated at 4*a* (0,*y*,*z*) Wyckoff sites, while Br1 and Br2 atoms are placed at 8*b* (*x*,*y*,*z*) and 4*a* sites, respectively. C and N atoms are also located at 4*a* positions. Figure 2 illustrates the quality of the fit from SXRD data, and Figure 3 displays two views of the crystal structure, including displacement ellipsoids for Cd and Br atoms. Figure 3b highlights the face-sharing of CdBr_6_ octahedra along the *c* axis. Table 1 contains the main crystallographic parameters after the Rietveld refinement.

In comparison with previous results [19], a subtle increase in the unit-cell parameters is observed in the present sample. This volume expansion can be associated with the presence of structural defects (vacancies), as it was observed previously in both hybrid and all-inorganic halide systems [24,25]. Additional refinements considering atomic vacancies did not lead to an improvement of the discrepancy factors. Considering that the volume cell increase is small (≈0.4%) it is possible to infer that the vacancy level is also low. These structural differences also are observed in the Cd-Br distances, as illustrated in Table 2. The distance increment mainly affects the Cd–Br2 bonds, which allows supposing that this bromine site is more plausible to present vacancies.

**Figure 4:**
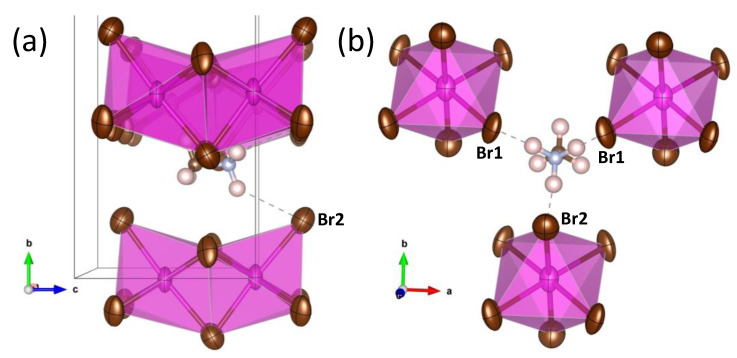
Views of MA with the expected hydrogen positions and H-bond interactions. (**a**) along a-axis direction, (**b**) along c-axis direction.

With the collected data (both angular resolution and high Q range), it is possible to reasonably refine anisotropic displacement parameters (ADP). The displacement factors of Br atoms are quite anisotropic, as shown in Figure 3, and they display a flattened shape (oblate type) with the disks perpendicular to the Cd-Br chemical bonds. In this configuration, quite standard in perovskites, the thermal vibrations are allowed in the perpendicular plane to the covalent Cd-Br bonding. The anisotropy of Br1 is much superior, with r.m.s. (root mean square) displacements of 0.11 Å parallel to the chemical bond and 0.30 Å and 0.22 Å perpendicular to it (for Br2, r.m.s.’s are 0.23 Å, 0.25 Å and 0.26 Å, respectively). As also shown in Figure 3, CH_3_NH_3_^+^ groups are in the space between the chains, roughly aligned along **c** axis. H atoms could not be localized from SXRD data. However, the MA position and the r.m.s. displacements suggest that the H-bond interaction of MA is stronger with Br2 atoms. To stand out this, the H atoms were added considering: (a) the expected distances and angles for the MA molecule and (b) the closeness with bromine atoms, with the aim to visualize the probable H-bond interactions. Figure 4 shows the environments of a MA unit, and the dashed lines highlight the H···Br distances less than 3 Å. This figure reveals that the disk-like ADP bromine is perpendicular to the H∙∙∙Br directions, hence, it is possible to speculate that the particular distribution of ADP parameters is a result of N–H···Br interactions. Finally, a highly polar character is expected for this compound, since all the CH_3_NH_3_^+^ units lie along the same direction, giving the acentric nature of the space group.

### 3.3. Optical Gap by UV-Vis Spectra and Photocurrent Properties

**Figure 5:**
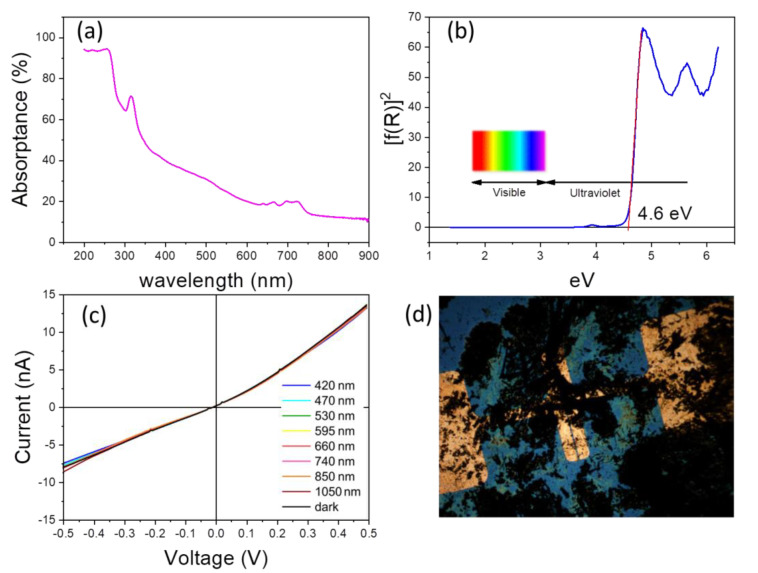
(**a**) Absorptance vs. wavelength of the incident radiation and (**b**) Kubelka–Munk transformed diffuse reflectance spectrum for CH_3_NH_3_CdBr_3_. (**c**) Photocurrent measurements in 420–1050 nm wavelength range and (**d**) optical image of optoelectronic device.

The absorption capacity of MACdBr_3_ powder prepared by mechano-chemistry was investigated by diffuse reflectance UV/vis spectroscopy. Figure 5a shows the UV-vis absorption spectrum, where there are two relative maximum absorption regions placed in the ranges 300–330 and 700–740 nm. The slope disruption at 300–330 nm corresponds to an exciton absorption peak that reveals the UV light activity of the sample; this exciton peak is due to the formation of an electron-hole pair that is favoured in nanostructured samples, containing nanoplates, as observed here (Figure 1) [26,27]. The onset at ~750 nm shows the absorption by minor MABr impurities [28].

Figure 5b illustrates the optical absorption coefficient related to the Kubelka–Munk function (F(R) = (1 − R)^2^/2R), being R the reflectance of each sample, vs. wavelength in eV. The band gap has been calculated by extrapolating the linear region to the abscissa. The value obtained for MACdBr_3_ (~4.6 eV; ~270 nm) is shifted to the UV region in comparison to the corresponding lead composition (MAPbBr_3_), which presents a band gap around 2.2 eV [29]. This shift will hamper its use in photovoltaic devices but could make this material useful for optical applications with short-wavelength UV radiation.

The optoelectronic properties of this phase were evaluated from photocurrent measurements. Figure 5c,d show the I vs. V curves at different wavelengths and an image of the used device, respectively. Figure 5c plots a typical ohmic behavior and display a negligible effect of illumination at different energies within the visible light range. This result confirms that this system is not an appropriate material for solar cells technology. However, as was mentioned above, it may find applications in the ultraviolet spectra range.

## 4. Conclusions

In contrast with the well-known MAPbBr_3_ hybrid perovskite, structurally consisting of a 3D framework of corner-sharing PbBr_6_ octahedra, the Cd counterpart contains infinite chains of face-sharing CdBr_6_ octahedra, with the CH_3_NH_3_^+^ units lying in between. The acentric nature of the *Cmc*2_1_ space group implied that the organic units are all aligned along the same direction, conferring a polar character to this compound. Starting from the obtained atomic positions and anisotropic displacement factors for Cd, Br, C and N, it was possible propose the H positions, unveiling the H-bond interactions between the CH_3_NH_3_^+^ units and CdBr_6_ octahedral chains. The different structural arrangement is a consequence of the much smaller size of Cd^2+^ vs. Pb^2+^, implying a tolerance factor greater than unity for the hypothetical perovskite. The large band gap and photocurrent results disable this material for solar cell applications, but it may find interest as a UV detector.

## Figures and Tables

**Table 1:** Structural parameters for CH_3_NH_3_CdBr_3_ from the Rietveld refinement in the orthorhombic *Cmc2*_1_ (No 36) space group, with *a* = 7.91722(5) Å, *b* = 13.7108(1) Å, *c* = 6.89374(2) Å, V = 748.33(1) Å^3^, Z = 4, from SXRD data at 295 K.

Atoms	*Wyckoff Sites*	*x*	*y*	*z*	*U*_iso_*/*U*_eq_	*Occ*
Cd	4*a*	0	0.0036(3)	1	0.035(3)	1
Br1	8*b*	0.2627(7)	0.4199(6)	0.248(3)	0.051(5)	1
Br2	4*a*	0	0.1612(7)	0.262(2)	0.056(9)	1
C	4*a*	0	0.32(1)	0.67(2)	0.12(1) *	1
N	4*a*	0	0.317(8)	0.85(2)	0.078(8) *	1
	*U*_11_	*U*_22_	*U*_33_	*U*_12_	*U*_13_	*U*_23_
Cd	0.029(3)	0.058(5)	0.017(1)	0	0	−0.001(5)
Br1	0.034(4)	0.082(6)	0.038(6)	−0.018(3)	−0.012(3)	0.002(5)
Br2	0.057(6)	0.063(7)	0.05(1)	0	0	0.011(7)
Discrepancy factors: R_p_: 8.46%; R_wp_: 11.6%; χ^2^: 4.27; R_Bragg_: 6.09%; MA_2_CdBr_4_: 16.7(2)%*w*/*w*						

The symbol * is indicated in the heading Uiso*.

**Table 2:** Atomic distances for CH_3_NH_3_CdBr_3_ from the Rietveld refinements in comparison with those reported by Hassen et al.

	Present Structure	Hassen et al. [19]
Cd–Br1	2.765 Å	2.774 Å
Cd–Br1	2.787 Å	2.783 Å
Cd–Br2	2.816 Å	2.769 Å
Cd–Br2	2.792 Å	2.786 Å

## Data Availability

Not applicable.

## Supplementary Materials

The following are available online at https://www.mdpi.com/article/10.3390/ma14206039/s1, Figure S1: EDX spectrum and quantitative results for MACdBr_3_ obtained with 18 kv of acceleration potential. H, N and C could not be observed in the presence of heavy Cd and Br atoms. The theoretical Cd/Br ratio of 1:3 is close to that found of 26.32%/73.68%, Figure S2: FE-SEM images of MACdBr_3_ with different magnifications, illustrating the overall aspect of this material obained by ball milling.

## References

[1] Grätzel M. (2014). The light and shade of perovskite solar cells. Nat. Mater..

[2] Green M.A., Ho-Baillie A., Snaith H. (2014). The emergence of perovskite solar cells. Nat. Photon.

[3] Snaith H.J. (2013). Perovskites: The Emergence of a New Era for Low-Cost, High-Efficiency Solar Cells. J. Phys. Chem. Lett..

[4] Park N.-G. (2013). Organometal Perovskite Light Absorbers toward a 20% Efficiency Low-Cost Solid-State Mesoscopic Solar Cell. J. Phys. Chem. Lett..

[5] Etgar L., Gao P., Xue Z., Peng Q., Chandiran A.K., Liu B., Nazeeruddin K., Grätzel M. (2012). Mesoscopic CH_3_NH_3_PbI_3_/TiO_2_ Heterojunction Solar Cells. J. Am. Chem. Soc..

[6] Kim H.-S., Lee C.-R., Im J.-H., Lee K.-B., Moehl T., Marchioro A., Moon S.-J., Humphry-Baker R., Yum J.-H., Moser J.-E. (2012). Lead Iodide Perovskite Sensitized All-Solid-State Submicron Thin Film Mesoscopic Solar Cell with Efficiency Exceeding 9%. Sci. Rep..

[7] Hsu H.-Y., Ji L., Ahn H.S., Zhao J., Yu E.T., Bard A.J. (2015). A Liquid Junction Photoelectrochemical Solar Cell Based on p-Type MeNH_3_PbI_3_ Perovskite with 1.05 V Open-Circuit Photovoltage. J. Am. Chem. Soc..

[8] Kojima A., Ikegami M., Teshima K., Miyasaka T. (2012). Highly Luminescent Lead Bromide Perovskite Nanoparticles Synthesized with Porous Alumina Media. Chem. Lett..

[9] Gao P., Grätzel M., Nazeeruddin M.K. (2014). Organohalide lead perovskites for photovoltaic applications. Energy Environ. Sci..

[10] Saparov B., Mitzi D.B. (2016). Organic–Inorganic Perovskites: Structural Versatility for Functional Materials Design. Chem. Rev..

[11] Zhang M., Yu H., Lyu M., Wang Q., Yun J.-H., Wang L. (2014). Composition-dependent photoluminescence intensity and prolonged recombination lifetime of perovskite CH_3_NH_3_PbBr_3_−xClxfilms. Chem. Commun..

[12] Alvarez-Galván M.C., Alonso J.A., López C.A., Lopez-Linares E., Contreras C., Lazaro M.J., Fauth F., Huerta M.V.M. (2018). Crystal Growth, Structural Phase Transitions, and Optical Gap Evolution of CH_3_NH_3_Pb(Br1–xClx)_3_ Perovskites. Cryst. Growth Des..

[13] Lopez C.A., Alvarez-Galvan M.C., Huerta M.V.M., Fernandez-Diaz M.T., Alonso J.A. (2019). Dynamic Disorder Restriction of Methylammonium (MA) Groups in Chloride-Doped MAPbBr 3 Hybrid Perovskites: A Neutron Powder Diffraction Study. Chem. A Eur. J..

[14] López C.A., Alvarez-Galván M.C., Martínez-Huerta M.V., Fauth F., Alonso J.A. (2020). Crystal structure features of CH_3_NH_3_PbI_3_−xBrx hybrid perovskites prepared by ball milling: A route to more stable materials. CrystEngComm.

[15] Dirin D., Dreyfuss S., Bodnarchuk M., Nedelcu G., Papagiorgis P., Itskos G., Kovalenko M.V. (2014). Lead Halide Perovskites and Other Metal Halide Complexes As Inorganic Capping Ligands for Colloidal Nanocrystals. J. Am. Chem. Soc..

[16] Li Y., Yang K. (2019). High-throughput computational design of organic–inorganic hybrid halide semiconductors beyond perovskites for optoelectronics. Energy Environ. Sci..

[17] Koliogiorgos A., Baskoutas S., Galanakis I. (2017). Electronic and gap properties of lead-free perfect and mixed hybrid halide perovskites: An ab-initio study. Comput. Mater. Sci..

[18] Kallel A., Mlik Y., Courseille C., Couzi M. (1992). Structural phase transition in single-crystal CH_3_NH_3_CdBr_3_: I. Experimental studies. J. Phys. Condens. Matter.

[19] Ben Hassen R., Ben Salah A., Kallel A., Daoud A., Jaud J. (2002). Crystal structure of monomethylammonium tribromocadmate(II). J. Chem. Crystallogr..

[20] Vassilakopoulou A., Papadatos D., Koutselas I. (2018). Polystyrene based perovskite light emitting diode. Appl. Mater. Today.

[21] Fauth F., Boer R., Gil-Ortiz F., Popescu C., Vallcorba O., Peral I., Fullà D., Benach J., Juanhuix J. (2015). The crystallography stations at the Alba synchrotron. Eur. Phys. J. Plus.

[22] Rietveld H.M. (1969). A profile refinement method for nuclear and magnetic structures. J. Appl. Crystallogr..

[23] Rodríguez-Carvajal J. (1993). Recent advances in magnetic structure determination by neutron powder diffraction. Phys. B Condens. Matter.

[24] López C.A., Abia C., Rodrigues J.E.F.S., Serrano-Sánchez F., Nemes N.M., Martinez J.L., Fernandez-Díaz M.T., Biškup N., Alvarez-Galván C., Carrascoso F. (2020). Enhanced stability in CH_3_NH_3_PbI_3_ hybrid perovskite from mechano-chemical synthesis: Structural, microstructural and optoelectronic characterization. Sci. Rep..

[25] López C.A., Abia C., Alvarez-Galván M.C., Hong B.-K., Martínez-Huerta M.V., Serrano-Sánchez F., Carrascoso F., Castellanos-Gómez A., Fernández-Dıaz M.T., Alonso J.A. (2020). Crystal Structure Features of CsPbBr_3_ Perovskite Prepared by Mechanochemical Synthesis. ACS Omega.

[26] Watthage S. (2017). Solution-Processed Fabrication of Hybrid Organic-Inorganic Perovskites & Back Interface Engineering of Cadmium Telluride Solar Cells, University of Toledo. http://rave.ohiolink.edu/etdc/view?acc_num=toledo1512390043951256.

[27] Khokhra R., Bharti B., Lee H.-N., Kumar R. (2017). Visible and UV photo-detection in ZnO nanostructured thin films via simple tuning of solution method. Sci. Rep..

[28] Yang M., Zhang T., Schulz P., Li Z., Li G., Kim D.H., Guo N., Berry J.J., Zhu K., Zhao Y. (2016). Facile fabrication of large-grain CH_3_NH_3_PbI_3_−xBrx films for high-efficiency solar cells via CH_3_NH_3_Br-selective Ostwald ripening. Nat. Commun..

[29] López C.A., Martínez-Huerta M.V., Alvarez-Galván M.C., Kayser P., Gant P., Castellanos-Gomez A., Fernández-Díaz M.T., Fauth F., Alonso J.A. (2017). Elucidating the Methylammonium (MA) Conformation in MAPbBr_3_ Perovskite with Application in Solar Cells. Inorg. Chem..

