# Supplementary material for: Mechano-Chemical Synthesis, Structural Features and Optical Gap of Hybrid CH3NH3CdBr3 Perovskite
Source: Materials (Basel). 2021 Oct 13;14(20):6039. doi: 10.3390/ma14206039 (PMC8538067; doi:10.3390/ma14206039)
Supplement: Supplementary file 1 [file materials-14-06039-s001.zip › materials-1374480-supplementary.pdf]

Supplementary Information

Mechano-Chemical Synthesis, Structural Features and Optical Gap of Hybrid $CH_3NH_3CdBr_3$ Perovskite

José Antonio Alonso [1,*], Paula Kayser [1,2], Bo-Kyung Hong [1,3], María Consuelo Álvarez-Galván [3], Francois Fauth [4] and Carlos Alberto López [5,*]

1 Instituto de Ciencia de Materiales de Madrid, Consejo Superior de Investigaciones Científicas, Cantoblanco 28049 Madrid, Spain; Pakayser@ucm.es (P.K.); sally.bkhong@ies.upm.es (B.-K.H.)
2 Departamenton de Química Inorgánica, Facultad de Ciencias Químicas, Universidad Compluense de Madrid, 28040 Madrid, Spain
3 Instituto de Catálisis y Petroleoquímica, Consejo Superior de Investigaciones Científicas, Cantoblanco 28049 Madrid, Spain; c.alvarez@icp.csic.es
4 CELLS–ALBA synchrotron Light Facility, Cerdanyola del Valles, Barcelona, E-08290, Spain; ffauth@cells.es
5 Instituto de Investigación en Tecnología Química (UNSL-CONICET) and Facultad de Química, Bioquímica y Farmacia, Universidad Nacional de San Luis, Almirante Brown 1455, San Luis, 5700, Argentina
* Correspondence: ja.alonso@icmm.csic.es (J.A.A.); calopez@unsl.edu.ar (C.A.L.)

Citation: Alonso, J.A.; Kayser, P.; Hong, B.-K.; Álvarez-Galván, M.C.; Fauth, F.; López, C.A. Mechano-Chemical Synthesis, Structural Features and Optical Gap of Hybrid $CH_3NH_3CdBr_3$ Perovskite. *Materials* **2021**, *14*, x. Nhttps://doi.org/10.3390/xxxxx

Academic Editor: Marc Cretin; Sophie Tingry; Zhenghua Tang

Received: 25 August 2021
Accepted: 8 October 2021
Published: date

Publisher's Note: MDPI stays neutral with regard to jurisdictional claims in published maps and institutional affiliations.

Copyright: © 2021 by the authors. Submitted for possible open access publication under the terms and conditions of the Creative Commons Attribution (CC BY) license (https://creativecommons.org/licenses/by/4.0/).

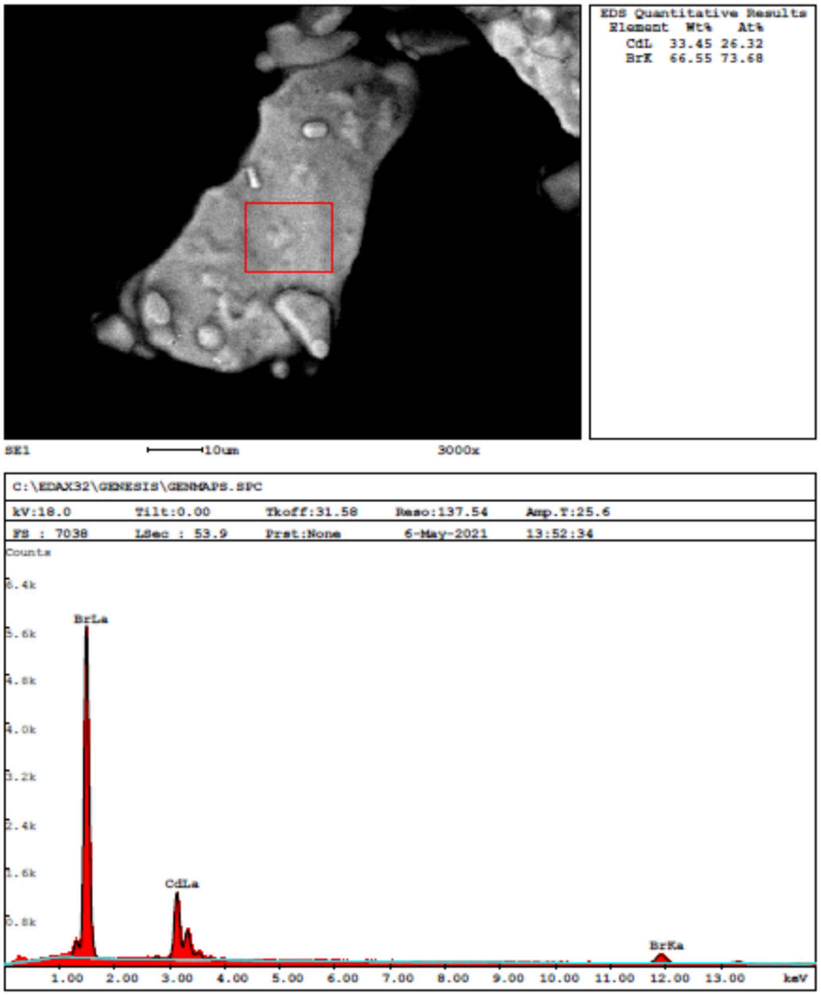

Figure S1. EDX spectrum and quantitative results for $MACdBr_3$ obtained with 18 kv of acceleration potential. H, N and C could not be observed in the presence of heavy Cd and Br atoms. The theoretical Cd/Br ratio of 1:3 is close to that found of 26.32%/73.68%.

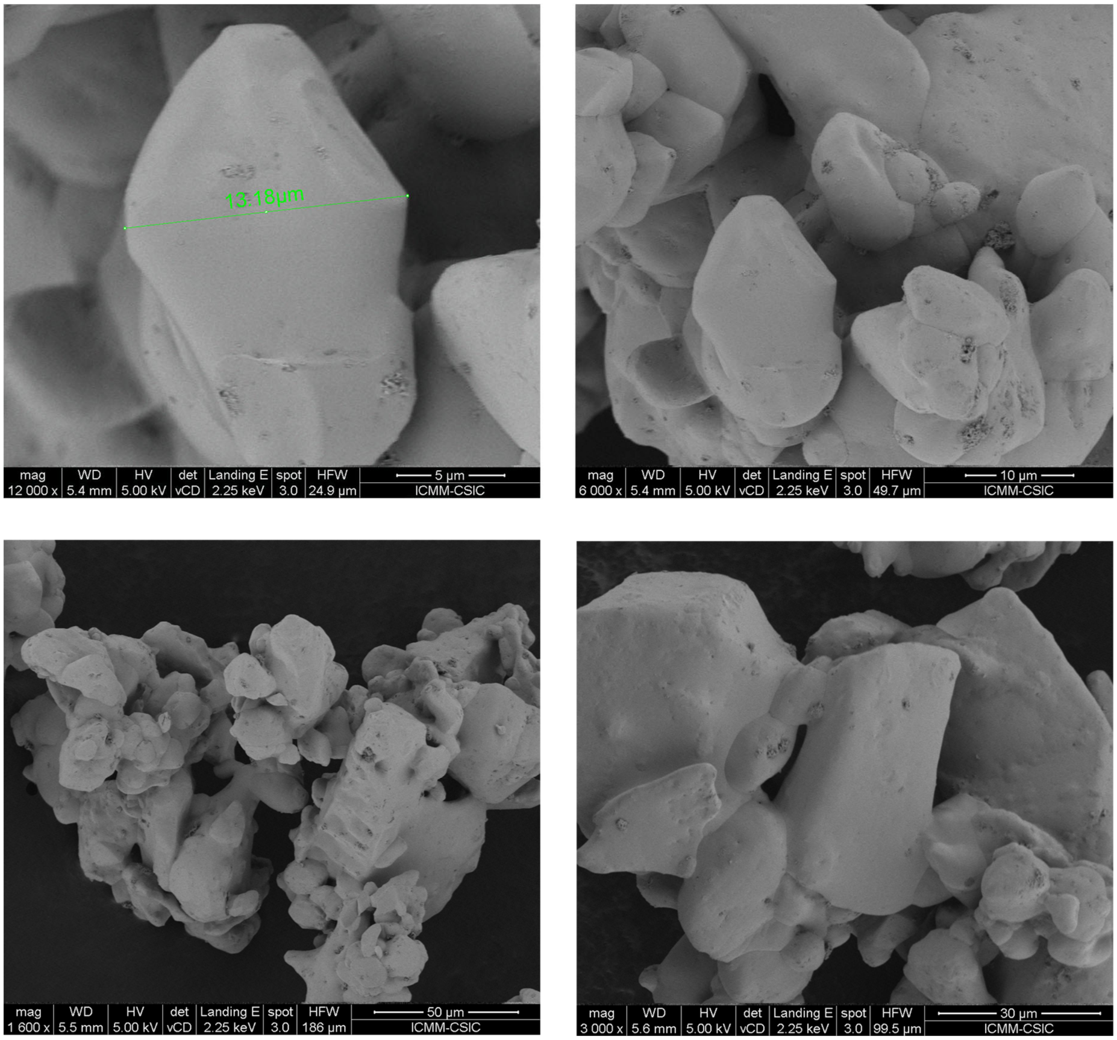

Figure S2. FE-SEM images of $MACdBr_3$ with different magnifications, illustrating the overall aspect of this material obained by ball milling.
